# Supplementary figures and images for: HP-CagA+ Regulates the Expression of CDK4/CyclinD1 via reg3 to Change Cell Cycle and Promote Cell Proliferation
Source: Int J Mol Sci. 2019 Dec 28;21(1):224. doi: 10.3390/ijms21010224 (PMC6981641; doi:10.3390/ijms21010224)

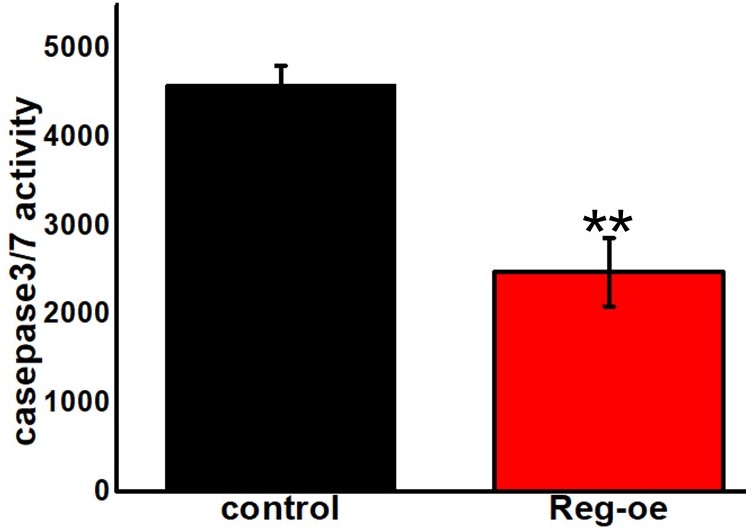

Supplement: Supplementary file 1 [file ijms-21-00224-s001.zip › sup figure/suppument1.JPG]

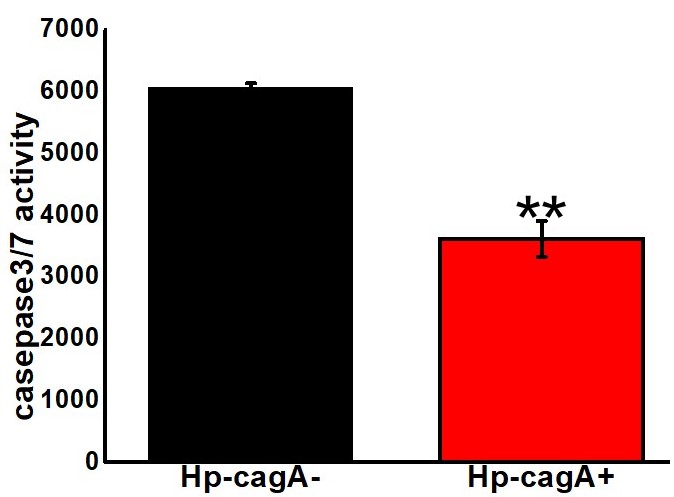

Supplement: Supplementary file 1 [file ijms-21-00224-s001.zip › sup figure/suppument2.JPG]
